# Assessment of Essential and Potentially Toxic Elements in Water and Sediment and the Tissues of *Sciaena deliciosa* (Tschudi, 1846) from the Coast of Callao Bay, Peru

**DOI:** 10.3390/toxics12010068

**Published:** 2024-01-14

**Authors:** Angélica Guabloche, Lorena Alvariño, Thiago Machado da Silva Acioly, Diego Carvalho Viana, José Iannacone

**Affiliations:** 1 Laboratorio de Ecología y Biodiversidad Animal (LEBA), Grupo de Investigacion de Sostenibilidad Ambiental (GISA), Facultad de Ciencias Naturales y Matemática, Universidad Nacional Federico Villarreal, Lima 15007, Peru; aguabloche@unfv.edu.pe (A.G.); lorenaalvarino@gmail.com (L.A.); 2 Postgraduate in Animal Science (PPGCA/UEMA), State University of Maranhão, São Luís 65081-400, Brazil; tmsacioly@gmail.com (T.M.d.S.A.); diego_carvalho_@hotmail.com (D.C.V.); 3 State University of the Tocantina Region of Maranhão (UEMASUL), Imperatriz 65900-000, Brazil; 4 Laboratorio de Ingeniería Ambiental, Coastal Ecosystems of Peru Research Group (COEPERU), Facultad de Ciencias Ambientales, Universidad Científica del Sur, Lima 150142, Peru; 5 Laboratorio de Zoología, Grupo de Investigación “One Health”, Facultad de Ciencias Biológicas, Universidad Ricardo Palma, Lima 150140, Peru

**Keywords:** biomonitoring, environmental impact, pollutant, food safety, heavy metals

## Abstract

The lorna drum *Sciaena deliciosa* is a coastal demersal species and one of the underlying artisanal fisheries in some areas of Peru, and is also a source of protein for Peruvian coastal dwellers. The investigation addresses concern about the environmental impact on this fish species and the potential risks to human health through the consumption of contaminated seafood. This research endeavors to assess the concentration of potentially toxic and essential elements in the muscle and liver tissues of *S. deliciosa*, in addition to the presence thereof in water and sediment capture areas on the coast of Callao, Peru. The study revealed that, in water samples, Ag, Ni, and Zn exceed Peruvian standards, but were below international standards, and Ba, P, Se, and Sn exceed international standards. In the sediments, As, Cd, Pb, Fe, and Zn were above international standards. In the fish, *S. delicious* muscle demonstrated As, Hg, and Pb exceeding at least one international standard. In the liver, As, Hg, Pb, and Cu exceed international standards. The study approach increased accuracy in risk assessments, offering crucial insights into the interplay between heavy metal pollution, water quality, and animal health, informing risk management strategies. Future studies can explore the long-term effects of heavy metal exposure on different organisms and consider their cumulative impact on health.

## 1. Introduction

The seas and oceans, covering 70% of the Earth’s surface, are one of humanity’s great hopes to ensure future food supply [1]. However, these ecosystems are constantly threatened by contamination due to the presence of anthropogenic pollutants, whose diversity varies according to the productive and urban activities taking place in the region. Environmental pollutants, such as heavy metal contamination in water sources, especially groundwater and surface water, have various health impacts on humans, such as liver failure, kidney damage, gastric and skin cancer, mental disorders, and negative effects on the reproductive system [2].

Potentially toxic elements like metals, metalloids, and non-metals are chemical elements of environmental interest due to the repercussions they have on different aquatic environmental components upon their presence. This type of pollution represents a major environmental challenge, mainly due to the characteristics of metals such as mobility, bioaccumulation, and molecular resistance [3]. These elements can bioaccumulate and biomagnify along the aquatic food chain through a variety of pathways, including respiration, adsorption, and ingestion [4,5]. The biomagnification phenomenon of potentially toxic elements in fish up the food chain is of interest to several studies [6,7,8]; since this ability can pose animal or human health risks through toxicity [9,10,11].

Elements lacking essential roles in metabolism, such as mercury (Hg), cadmium (Cd), lead (Pb), and aluminum (Al), are among the most prevalent toxicants exerting harm in the human body [12,13,14]. However, it is important to highlight that the class of PTEs also includes essential elements, with a role in human metabolism, such as copper (Cu), iron (Fe), nickel (Ni), zinc (Zn), and chromium (Cr) [14,15,16]. This study also indicates that there is a possibility of interaction between elements, highlighting that the presence of toxic metals can influence the action of essential elements [16]. This phenomenon can result in toxicity by interfering with metabolic processes.

The presence of these elements in natural aquatic ecosystems can cause algal proliferation, oxygen deficiency, and even the death of aquatic organisms [3,15,17]. PTEs can impact directly by entering aquatic living organisms and inducing toxicity or exert indirect effects by disrupting the intricacies of the food chain, leading to cascading effects on the overall ecological balance of aquatic environments [12,14,18,19,20]. Studies indicate that arsenic (As) can cause visceral cancer and liver and bladder cancer [20,21]; while, cadmium (Cd) can cause a decline in cognitive capacity, kidney disorders, and consequently, bone loss [22,23,24]; and lead (Pb) can affect the central nervous system (CNS), causing hyperactivity, fatigue, anemia, and decreased IQ [25,26]. Furthermore, the International Agency for Research on Cancer (IARC) classifies As, Cd, and Cr as Group 1 Carcinogenic to Humans [27].

Fishes have been widely used as bioindicators of metals, metalloids, and non-metals contamination; moreover, they are an important part of the human diet due to their high protein content, low saturated fats, and sufficient omega fatty acids, and are known to support good health [16,28]. Fish muscle tissue is most commonly used for analysis, as it is a significant tissue for metal storage and is the primary edible part of the fish [29,30]. Therefore, various studies have been conducted worldwide on the contamination of different fish species by heavy metals [31,32,33]. For example, recent studies provide specific information about potentially toxic elements (As, Cd, Cr, Hg, Pb, Se, etc.) in Callao Bay’s surface water, sediment, mollusk (*Thaisella chocolata*), and fish muscle (*Mugil cephalus*, *Odontesthes regia*, and *Sciaena deliciosa*) [34,35,36].

Callao Bay is strategically important from both an industrial and tourist perspective. It serves as a recreational space for a significant portion of the Chalaca population. However, it continually receives discharges of wastewater from a diverse range of industries, including the products of fisheries, human food consumption, chemicals, a major oil refinery, and one of the country’s main seaports, which encompasses various loading and unloading areas (minerals, hydrocarbons, etc.), as well as domestic and agricultural sewers [34]. Additionally, there are discharges from the Chillón and Rímac Rivers, carrying residues of pesticides, minerals, and other products from activities along their entire course, causing a severe impact on the receiving environment [37,38]. Several studies have investigated anthropogenic pollutants like PTEs from different fishery areas of Peru [39,40,41,42].

The Lorna *Sciaena deliciosa* (Tschudi, 1846) is a coastal demersal species taxonomically classified within the Sciaenidae family. It is one of the species that supports artisanal fishing in some areas of Peru and serves as a protein source for coastal residents in the country. Coastal species, mostly extracted by artisanal or smaller-scale fisheries, are liza (*Mugil cephalus*) and lorna drum (*Sciaena delicious*). The total catch of this group of species was stable at 43,000 tons [43]. This group is distributed from Puerto Pizarro (Peru) to Antofagasta (Chile) [44]. The importance of studying the specimens has been considered from the following perspectives: it is one of the species supporting artisanal fishing in some areas of Peru, it serves as a protein source for coastal residents in Peru, and it can store a higher concentration of contaminant compounds in its body compared to those present in the environment. Therefore, it is an important indicator of contamination, but this also implies that its consumption can become a health problem for populations relying on this resource.

In Peru, there is an increase and diversification of human activities that have led to environmental deterioration. As a consequence of these various activities, numerous discharges are released into the marine environment, often altering the quality of the physical and chemical parameters typical of the environment. This research endeavors to assess the concentration of potentially toxic and essential elements in the muscle and liver tissues of *S. deliciosa*, as well as the presence thereof in the water and sediments from the coastal zone of Callao Bay. The significance and originality of this study hold relevance not only locally but also contribute to the broader international academic discourse on environmental impacts and the innovative aspects of this investigation.

## 2. Materials and Methods

### 2.1. Study Area, Field Parameters, and Ethical Aspects

**Figure 1:**
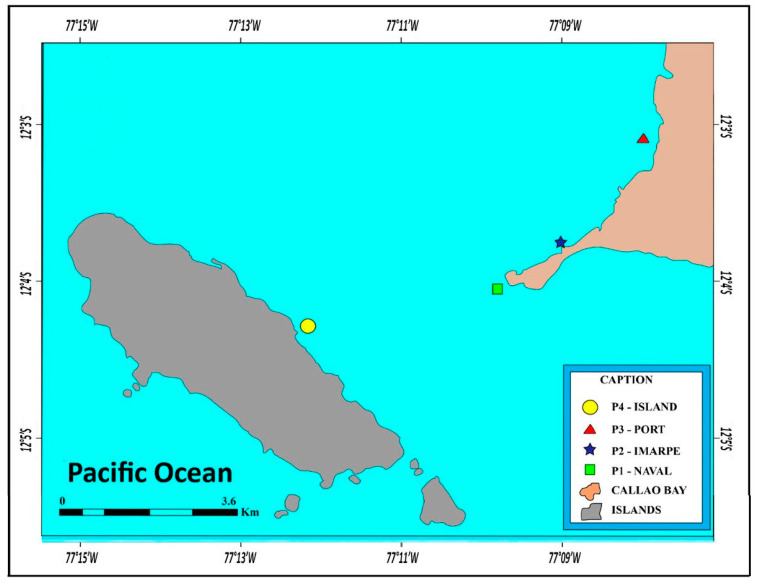
The geographic location of the sampling stations/areas in the coastal zone of Callao Bay, Peru. P1: the Naval School (12°4′3.30″ S; 77°10′8.60″ O); P2: Instituto Peruano do Mar, IMARPE (12°3′56.20″ S; 77°9′30.70″ O); P3: Callao Pier (12°2′34.10″ S; 77°9′15.40″ O); P4: San Lourenço Island (12°5′16.57″ S; 77°11′52.03″ O). This island and its surroundings have a very well-preserved biota due to the almost total absence of human activity for many years.

The study was conducted in the coastal zone of the Bay of Callao (Peru), where four sampling campaigns were carried out during the fall, winter, spring, and summer. To obtain the exact geographical location of each site, during the sample collection, the areas were georeferenced using a GPS and a GARMIN echo sounder model map 4215 and were plotted on a map using ArcGIS 10.3.1 and ArcMAP 10.3.1 software (Figure 1).

This study followed all the national and international ethical aspects of ecotoxicology and collected fishes. The number of fishes used was determined according to the principle of the three “r’s” [45]. For proper management of fish, reagents, and seawater samples, as well as their disposal, the “Safety Plan for Laboratories and Workshops (SSST-PLC-01)” followed Rectoral Resolution No. 10026-2009-UNFV and the “Security Protocol for Engineering, Architecture and Natural Sciences Laboratories and Workshops (SSST-PS-02)”.

The fish were acquired from fishing conducted by artisanal fishermen who presented the Artisanal Fishing Certificate for natural or legal persons with the Fishing Permit of the Maritime Port of Callao, Peru, so they did not need specific authorization to obtain the fish (Ministerial Resolution 409-2017-PRODUCE). *Sciaena deliciosa* is not on the list of hydrobiological resources with a closed season calendar according to PRODUCE regulations, so obtaining this biological material will not affect this marine fish in its natural environment.

To carry out this work, physicochemical parameters such as surface temperature, pH, salinity, total dissolved solids, conductivity, dissolved oxygen, ammonium, nitrates, turbidity, chlorophyll oxide reduction potential, and phycocyanins were taken in situ with an EXO 2 multiparameter. Chlorophyll and phycocyanins were measured using the algae torch fluorometric method. These parameters are only used to guarantee the quality of water for human consumption and to protect aquatic ecosystems.

### 2.2. Sample Collection: Water, Sediment, and Fish Tissue

For potentially toxic element analysis, 500 mL of water was collected in washed and nitric acid-treated polyethylene containers (tripled per point). The pH was adjusted to 2 with the same acid, and the samples were kept at 4 °C until being transported in a cooler for subsequent analysis. Additionally, samples of 500 g of surface sediments in the study area were obtained using a Van Veen grab with a sweeping area of 0.04 m^2^, launched from the boat. Only the central part of the sediment was collected to avoid possible contamination from the grab walls, and it was stored in plastic bags, properly labeled, and kept in a cold chain until reaching the Ecology and Animal Biodiversity Laboratory.

The fishes were captured using three-gill nets with a mesh size of 2 1/4 inches, a thread size of 40, measuring 60 m in length by 6 m in height. Evisceration was performed on-site, selecting the liver and muscle tissues. Muscle tissue cuts were made above the lateral line and at the level of the beginning of the dorsal fin using a clean scalpel, and they were grouped into pools. The livers of each fish were also grouped into pools, and both tissues were placed in polyethylene bags and frozen at −4 °C for preservation and transport. Later, at the Ecology and Animal Biodiversity Laboratory of the Universidad Nacional Federico Villarreal (UNFV), they were stored at −20 °C for subsequent analysis.

A total of 30 live fish were collected with the help of residents and fishermen from the coastal area of Callao Bay, Peru. The collected fish species was “Lorna drum” *Sciaena deliciosa* (Tschudi, 1846) (total length (TL): 21.26 ± 1.60, 19–25; weight (W): 143.83 ± 31.08, 96.05–208). This fish species was selected due to its regular consumption by the local population and its various niches in the aquatic ecosystem.

### 2.3. Determination of Potentially Toxic and Essential Elements in Water, Sediments, and Tissues

The contents of potentially toxic elements (aluminum—Al, antimony—Sb, arsenic—As, barium—Ba, cadmium—Cd, chromium—Cr, mercury—Hg, lead—Pb, lithium—Li, nickel—Ni, strontium—Sr, titanium—Ti, and silver—Ag) and essential elements (beryllium—Be, boro—B, selenium—Se, sílice—SI, phosphorus—P, copper—Cu, iron—Fe, calcium—Ca, cerium—Ce, potassium—K, magnesium—Mg, manganese—Mn, molybdenum—Mo, sodium—Na, vanadium—Va, cobalt—Co, tin—Sn, and zinc—Zn) were determined using inductively coupled plasma optical emission spectrometry (SHIMADZU, ICPE-9000) following the analytical methodology of the U.S. Environmental Protection Agency (US EPA), the American Public Health Association (APHA), the American Water Works Association (AWWA), and the Water Environment Federation (WEF), which varied according to the analysis source. For seawater, EPA method 200.7 was employed. An aliquot of the water sample was measured and digested with nitric and hydrochloric acid. The Chelation extraction technique was then applied using APDC (ammonium pyrrolidine dithiocarbamate) and MIBK (methyl isobutyl ketone) [46].

To determine the total element concentrations in the sediment, a one-gram sample (dry weight) was taken and mixed with a 6:2 mixture of nitric and hydrochloric acid for 2 h or until complete digestion [47,48]. The tissues were dried in an oven at 80 °C to obtain a constant weight, then pulverized and homogenized in a porcelain mortar, and stored in tightly sealed glass vials with Bakelite caps. Chemical digestion was performed with concentrated nitric acid, hydrochloric acid, and hydrogen peroxide. Calibration standards and blanks received the same treatment [49,50]. For Hg analysis, the samples were digested with nitric acid, hydrochloric acid, and potassium permanganate [51].

All element analyses were carried out in a laboratory with national and international accreditation for testing laboratories [52]. The accuracy and precision of the results of the samples were assessed using certified reference material of fish protein (DORM 4, Fish Protein Certified Reference Material for Trace Metals) supplied by the National Research Council Canada. The recovery rates for DORM-4 reference samples for Al, As, Cd, Ca, Cr, Cu, Fe, P, Se, Mg, Mn, Sr, Pb, Zn, and Hg ranged from 86% to 109%, thus confirming the accuracy of the analyses. Furthermore, the laboratory holds accreditation and acknowledgment from several reputable bodies, including IAS (International Accreditation Service), INACAL (National Quality Institute of Peru), and SENASA (National Agrarian Health Service). Additionally, it has received the Trinorma certification, meeting ISO 9001 [53], ISO 14001 [54], and ISO 45001 [55] standards, granted by AENOR (Spanish Association for Standardization and Certification).

### 2.4. Bioconcentration Factor (BCF)

The term used to quantify the tendency of a substance to concentrate in aquatic organisms is the bioconcentration factor (BCF). BCF = concentration of the substance in the body/concentration of the substance in water. Fish are normally the target of bioconcentration studies due to their importance as a food source for humans and the availability of standardized assays for these organisms. Thereby, a BCF of 1000 means that the organism concentrates the product up to a value a thousand times higher than that of the environment [49].

### 2.5. Risk Assessment: Estimated Daily Intake (EDI), Target Hazard Quotient (THQ), Hazardous Index (HI), and Carcinogenic Risk (CR)

The calculation of estimated daily intake (EDI) for the meal size of seafood was performed from fish muscles. The risk factors and permissible consumption range were calculated by following [56].

EDI = C × IR/BW

where:

C = the concentration of metal present in the body parts (muscle) (mg/kg) (mg.kg^−1^);

IR = the daily ingestion rate in Peru = 50 g/day [57];

BW = body weight (70 kg for Peruvian adults and 15 kg for Peruvian children) [58].

The target hazard quotient (THQ) is the estimation of the non-carcinogenic level due to the exposure to pollutants. If the value of THQ is below 1, it means that there is no possible health threat, whereas if the value of THQ is ≥1, it means that there is a possible health threat and that corrective measures should be taken [59]. The THQ index can be calculated using the equation below [56]:

THQ = (EFr × ED × FiR × C/RfD × BW × AT) × 10^−3^

where:

EFr = the total exposure frequency, which is equivalent to 365 days/year;

ED = the exposure duration (75 years for adults/6 years for children);

FiR = the rate of fish ingestion (g/day);

C = the mean concentration of trace elements in foodstuff (mg/kg);

RfD = the oral reference dose according to the USEPA (mg/g/day);

BW = the mean of body weight (for a grown person it is assumed to be 70 kg; for children it is assumed to be 15 kg);

AT = the mean of exposure time (365 days per year × ED);

The oral reference dose (RfD) for the trace element As is 0.0003 mg/kg/day, for Cd it is 0.001 mg/kg/day, for Cr it is 0.0015 mg/kg/day, for Cu it is 0.40 mg/kg/day, for Fe it is 0.8 mg/kg/day, for Hg it is 0.0005 mg/kg/day, for Mn it is 0.14 mg/kg/day, for Ni it is 0.02 mg/kg/day, for Se it is 0.005 mg/kg/day, for Ag it is 0.005 mg/kg/day, for Pb it is 0.004 mg/kg/day, and for Zn it is 0.3 mg/kg/day.

The hazardous index (HI) is the summative of the THQ for all trace elements to which an individual might be exposed. The HI was calculated using the equation below [60]. Avalue of HI ≥ 1 reveals the possible non-carcinogenic risk to humans.

HI = Σ THQ = THQ (iAs) + THQ (Cr) + THQ (Cu) + THQ (Fe) + THQ (Mn) + THQ (Pb) + THQ (Zn) + THQ (Hg) + THQ (Ni) + THQ (Se) + THQ (Ag) + THQ (Cd)

The carcinogenic risk (CR) index assesses the probability of an individual developing cancer over their lifespan due to exposure to carcinogenic metal(loid)s. The assessment of CR was conducted on Pb and As only, where the cancer slope factors (CSF) for Pb and As were 0.0085 mg/kg/day and 1.5 mg/kg/day, respectively [61]. However, the calculation of carcinogenic risk should be based on inorganic As (iAs), which is known to be more toxic [62]. Although the analysis of iAs was not conducted in this study, the carcinogenic risk calculation assumed that the iAs in the fish ranged from 1% to 10% of the total As [63]. Both authors used the maximum assumption percentage of iAs. Therefore, in this study, the iAs was assumed to be 10% of the total As. A value of CR ≤ 10^−6^ is considered an acceptable range, whereas a value of CR ≥ 10^−3^ is considered intolerable [64]. The calculation of CR was given by the following equation:

CR = CSF × EDI

where:

CSF = the cancer slope factor determined by the USEPA (mg/kg/day);

EDI = estimated daily intake.

### 2.6. Quality Assessment: National and International Standards

In the case of the water samples, the results were compared using the National Environmental Quality Standards (NEQS) for water according to Supreme Decree No. 015-2015-MINAM. They were also compared with international standards such as the EPA’s National Recommended Water Quality Criteria and the World Health Organization (WHO) Guidelines for Drinking Water Quality: Recommendations [65].

The assessment of sediment quality was conducted by comparing the results with international standards since there are no environmental quality standards for marine sediments in Peru yet. The criteria established by the WHO/FAO and the Codex Alimentarius Commission for metal content in tissues, were compared with international standards such as the Food and Agriculture Organization (FAO) Heavy Metal Regulations—Faolex. (Legal Notice No. 66/2003) [66,67].

### 2.7. Data Analysis

To assess significant differences among the average total toxic and essential element concentrations at sampling stations or within the same station for different samplings, analysis of variance (ANOVA) was utilized, assuming that the data follows a normal distribution and that the population variances are equal. Therefore, before applying the analysis, it was necessary to conduct the Kolmogorov–Smirnov normality test and Levene’s test for variance homogeneity. Additionally, to find the relationship between two variables, the Pearson correlation coefficient was employed. For all statistical analyses, the significance criterion was set at *p* < 0.05. To compare the means between points, the Tukey test was used. The data were analyzed using the statistical software SPSS version 22 at UEMASUL.

## 3. Results

### 3.1. Water Physicochemical Parameters, Presence of Nitrogen Compounds, and Concentration of Potentially Toxic and Essential Elements in Water

During the study period, the average surface temperature was 20.3 °C, with a minimum of 16.9 °C and a maximum of 24.6 °C, recorded in the months of August and May, corresponding to winter and autumn, respectively. The pH ranged from 7.9 to 9.1, with an average of 8.2, while salinity varied between 34.7 and 35.5 g/L. The presence of nitrogen compounds was also detected, with an average of 52.65 mg/L for ammonium (NH_4_^+^) and 510.83 mg/L for nitrate (NO_3_^−^). Other values of the water samples’ physicochemical variables are presented in Table 1.

The results obtained from the seawater samples collected from the four sampling campaign areas are presented in Table 2. The concentration values of arsenic (<0.009 mg/L), beryllium (<0.0003 mg/L), cerium (<0.0044 mg/L), cobalt (<0.0028 mg/L), tin (<0.0138 mg/L), mercury (<0.0001 mg/L), and cadmium (<0.0015 mg/L) were found to be below the detection limits (LD) of the spectrophotometer in most areas. The elevated levels of Ag, Ni, and Hg exceeded Peruvian standards, while for Pb they surpassed both the Peruvian standards and those established by the World Health Organization (WHO), highlighting significant concerns about the environment and human health (Table 2).

### 3.2. Concentration of Potentially Toxic and Essential Elements in the Sediment

Elevated concentrations of certain potentially toxic and essential elements, including cadmium (5.24 mg/kg), lead (122.95 mg/kg), and zinc (391.43 mg/kg), were observed in the sediment (Table 3). These elements have values exceeding standards for heavy metals in sediment [68].

### 3.3. Concentration of Potentially Toxic and Essential Elements in Muscle and Liver Tissues

The contents of the muscle tissue of *S. deliciosa* are shown in Table 4. Meanwhile, in Table 5, the concentrations of potentially toxic and essential elements in liver tissue are shown. For example, the lead concentration in the muscle tissue, measuring 3.78 mg/kg, exceeds the established international standards.

Antimony (<0.6 mg/kg), bismuth (<0.8 mg/kg), cerium (<0.74 mg/kg), cobalt (<0.64 mg/kg), molybdenum (<0.34 mg/kg), and vanadium (<0.18 mg/kg) were below the detection limits of the spectrophotometer. Nevertheless, the analysis of both tissues (muscular and hepatic) demonstrates that essential metals such as iron, copper, zinc, and manganese were found in higher concentrations. The potentially toxic elements that exceeded the previously discussed international standards were arsenic at 3.30 mg/kg, cadmium at 0.83 mg/kg, lead at 1.74 mg/kg, and nickel at 1.40 mg/kg (Table 5).

### 3.4. Bioconcentration Factor (BCF)

The bioconcentration study of lorna tissues revealed that, on average, it concentrated phosphorus 87,056 times more in the muscle than in the environment, iron (1350 times more), zinc (1330 times more), manganese (566 times more), arsenic (442 times more), and mercury (362 times more). Regarding the liver, the BCF showed that the elements with high values were phosphorus (42,493 times more), iron (3219 times more), zinc (3149 times more), copper (1882 times more), and mercury (1509 times more) (Table 6).

### 3.5. Risk Assessment: Estimated Daily Intake (EDI), Target Hazard Quotient (THQ), Hazardous Index (HI), and Carcinogenic Risk (CR)

The EDI values for adults were greater than the RfD for Cd, Cr, Hg, Pb, Ni, As, and Se. In the case of the values of THQ for the estimation of the non-carcinogenic level, these exceeded the value of 1 for Cd, Cr, Pb, As, and Se. Carcinogenic risk (CR) did not exceed the value of ≥10^−3^ for Pb and As (Table 7).

The EDI values for children were greater than the RfD for Cd, Cr, Hg, Ni, Pb, Ni, Ag, and As. In the case of the values of THQ for the estimation of the non-carcinogenic level, these exceeded the value of 1 for Pb, As, and Se. Carcinogenic risk (CR) did not exceed the value of ≥10^−3^ for Pb but exceeded the value for As (Table 8).

## 4. Discussion

The results of various studies indicate that Callao is one of the main areas on the Peruvian coast suffering from severe marine pollution [36,70,71]. Aquatic environments, especially rivers, and the sea, are the ultimate receptors of such contaminants/heavy metals, and slight changes in the quality of the environment, including physicochemical properties, can have a negative impact on the normal physiology of aquatic organisms, especially in fish, which are very sensitive to such changes [69,70,71,72,73]. The primary sources of contamination are domestic and industrial wastewater originating from the Rimac River, as well as maritime transportation activities due to the proximity of the port of Callao. This highlights the need for multidisciplinary studies in the area to generate updated technical information, ensuring environmental balance and food security for the population.

The process of mineral absorption in the digestive tract of fish involves the passage of mineral ions through the cell membranes of intestinal cells. Fish have different transport mechanisms for different minerals. For example, some minerals can be absorbed through active transport, where the cell uses energy to actively transport mineral ions against a concentration gradient. Once absorbed, minerals are transported through the bloodstream to various parts of the body, including the liver. The liver plays a crucial role in mineral regulation and metabolism. It stores, releases, and converts minerals as needed for the body’s metabolic and physiological functions [74]. It is important to note that different minerals may have different absorption and regulation pathways in the fish body. Furthermore, factors such as diet, water quality, and the specific characteristics of the fish species can significantly influence mineral absorption and metabolism.

The application of water quality indices revealed a substantial degradation in overall water quality due to the elevated presence of these metals. The innovative Monte Carlo simulation approach allowed for a more accurate assessment of health risks, considering the uncertainty associated with the data [75]. The results of this analysis revealed possible scenarios of human exposure to heavy metals and highlighted the areas of greatest concern, hence the critical need for effective interventions and regulations to mitigate heavy metal pollution and protect water quality. Based on the findings of our study, further action may be required to mitigate the risks associated with exposure to these substances. This could involve implementing measures to reduce the intake of these substances or address the sources of contamination.

The coastal marine area in Callao Bay, especially in the vicinity of the islands of San Lorenzo, El Frontón, and La Punta, exhibits geomorphological, sedimentary, and physicochemical characteristics that support the development and habitation of ecologically and economically significant marine species, either temporarily or permanently. These species are harvested by local fishermen. It is emphasized that, for low concentrations of metals in the water, the toxic properties thereof depend on ecological factors such as pH and organic ligands [76]. Furthermore, modifications to the physical factors of the environment (suspended solids, turbidity, color, temperature, etc.) may not be toxic but alter the physical characteristics of the water and affect aquatic biota. The monitoring of water, sediment, and biota may provide a comprehensive understanding of the overall health of water bodies within specific river basins [77].

In Callao, contamination by metallic elements stems from a variety of industries such as textiles, tanneries, paper mills, mining, and petrochemicals, whose wastewater discharges various potentially toxic metallic elements that are hazardous to the marine ecosystem and human health. In recent centuries, industrialization and globalization have had an impact on natural environments and their ability to support life [78]. Trace elements from anthropogenic sources have been shown to accumulate in the environment; in these cases, the local population is primarily exposed through the soil, water, food, air, commercial products, and occupational sources [79]. In turn, it has been observed that the concentration of heavy metals (Ni, Cr, Pb, Cu, Co, Zn, Fe, and Mn) in different fish tissues is several times higher than concentrations of these elements in the water [80].

Scholars have recorded levels of metals, metalloids, and non-metals in water, sediment, and *Thaisella chocolata* muscle from Callao’s Bay, and compared them with the maximum allowed limits set by national and international regulations [34]. In the present study, the average concentrations of As, Cd, Cu, Cr, Hg, Pb, and Se in all zones and stations exceeded the minimum concentration limit. The authors concluded that consuming specimens from the Bay of Callao would pose a severe health problem for people. Furthermore, oxidative stress arising from contact with potentially toxic elements can weaken the immune system, cause tissue damage, and even reduce the productive capacity of aquatic organisms.

In the current study, it was observed that the concentration of cadmium in the water samples barely reached values below the detection limits of the spectrophotometer (<0.0015 mg/L), while in the sediment, the values ranged between 1.45 and 9.53 mg/kg. This is because a significant portion of the cadmium entering aquatic surfaces from industrial effluents rapidly adsorbs into particulate matter and settles in sediments. It is noteworthy that the presence of potentially toxic metals in the water is due to industrial wastewater discharged into the sea through sewers, rivers, and channels. In this study, Pb was also found above the Peruvian standards.

It is known that marine sediments are commonly enriched with Zn, Cu, and Pb, which are also found in several ports and channels in the city [81]. In this study, these metals were found in concentrations higher than the permissible limits set by international standards only in areas P2 and P3, characterized by heavy vessel traffic and the mooring of boats of various sizes. The increase in Pb concentration in aquatic systems is primarily due to human activities such as transportation, fuel combustion, industrial contamination, transboundary transfer, urbanization, etc. [76].

Of all aquatic organisms, fish have historically been the first to be used to assess the effects of possible contaminants on these systems. This is because the fish community constitutes a prominent component of such ecosystems and also constitutes an important economic resource for humans. Their flesh has a high content of proteins and omega fatty acids, and low saturated fat content [1]. Moreover, fish are the only vertebrates whose life cycle is entirely aquatic, allowing the integration of the effects of exposure to toxic substances in that medium. This allows the extrapolation of their mechanisms of action with a higher degree of certainty for humans [82].

In the water samples, barium, selenium, cadmium, nickel, mercury, cobalt, silver, and phosphorus were above the permissible limits of (LP) at some sampling areas. The concentrations of potentially toxic metals in the water followed the following order: Ba > Se > Cd > Hg > Co > Ag > Ni > P; in the sediment samples: Al > Mg > Ni > Cr > B; in the muscle samples: K > P > Na > Ca > Fe > Zn > Al; and in the liver samples: K > P > Na > Mg > Ca > Cu > Sr > Mn. Regarding the sediment, elements like aluminum, magnesium, nickel, copper, and boron were found to be above the LP according to international standards in some of the sampling areas, mainly near the pier (P3) and IMARPE (P2).

In both tissues (muscle and liver), essential metals such as iron, copper, zinc, and manganese were found in higher concentrations, while non-essential and toxic metals such as arsenic, cadmium, mercury, nickel, and lead showed low concentrations. Overall, the quantity of potentially toxic metals showed little variability between the sampling areas, except for arsenic, lithium, and lead. The trace elements can be grouped into essential elements (Fe, Cu, Mn, Zn, etc.) and potentially toxic elements (As, Pb, Cd, Hg, etc.). However, it is important to highlight that all of them can pose a threat to the health of living organisms when they exceed the permissible limit, regardless of their classification [83].

In the Dhaleswari River in Bangladesh, a study revealed alarming levels of heavy metals, such as lead, cadmium, mercury, and arsenic, in fish samples collected in the area [84]. This bioaccumulation of heavy metals in aquatic species suggests possible environmental contamination in the river ecosystem, as is the case with the bioaccumulation detected in this study. These results indicate that lorna tissues, both in the muscles and the liver, can accumulate certain elements to a significant extent compared to their presence in the surrounding environment. The high bioconcentration factors suggest a potential environmental exposure or intake of these elements by lorna, leading to their accumulation in specific tissues. This information can be valuable for environmental monitoring and risk assessment, especially if the concentrations exceed safe levels.

The EDI values for adults and children were greater than the RfD for different substances (Cd, Cr, Hg, Ni, Pb, Ni, and As), this indicates that they may be exposed to levels of these substances that exceed the recommended limits, potentially posing a risk to their health. Based on these findings, further action may be required to mitigate the risks associated with the exposure to these substances. This could involve implementing measures to reduce the intake of these substances or addressing the sources. In terms of detectable levels of heavy metals, including lead, cadmium, and mercury in samples of foods intended for babies, in a study conducted by Amarh [85], the presence of these heavy metals is particularly worrying, considering the sensitivity of babies to toxic substances and their crucial stage of development. The need for regulatory intervention in and strict monitoring of the production and marketing of baby foods in Wa, Ghana, is evident to ensure food security and protect the health of the most vulnerable.

## 5. Conclusions

In the water samples, concentrations of Ag, Ni, and Zn exceeded the standards established in Peru, although they remain below international standards. On the other hand, Ba, P, Se, and Sn exceeded international standards. For the sediment samples, the concentrations of As, Cd, Pb, Fe, and Zn were above international norms. For the fish samples, the muscle of *S. delicious* demonstrates As, Hg, and Pb above at least one considered international norm, while in the liver samples, the most expressive values that exceeded international standards were those of As, Hg, Pb, and Cu.

Our approach has increased the accuracy of non-cancer and carcinogenic risk assessments, providing critical insights into the complex interplay between heavy metal pollution, water quality, and animal health. This research significantly informs risk management strategies. Future studies could explore the long-term effects of exposure to heavy metals on diverse organisms and consider the cumulative impact of multiple heavy metals on health.

## Figures and Tables

**Table 1:** Average, maximum, and minimum values of the physicochemical parameters in the waters of Callao Bay, Peru.

Parameters	Minimum	Maximum	Average	Standard Deviation
TEMP (°C)	16.90	24.60	20.26	1.76
PH	7.90	9.06	8.23	0.31
COND (mS/cm)	53.00	53.90	53.54	0.27
SAL (PPT)	34.70	35.50	35.07	0.22
TDS (g/L)	33,737.00	38,500.00	35,182.75	1852.58
LDO (mg/L)	0.65	4.32	2.04	0.98
TRANS (m)	0.75	7.50	3.18	1.68
TURB (NTU)	0.73	243.10	23.09	66.37
PHYCO (g/mL)	−106.00	0.10	−12.93	31.11
CHLORO (µG/L)	0.00	2.00	0.63	0.63
ORP (mV)	164.00	241.30	192.93	26.17
NH_4_^+^ (mg/L)	33.57	64.52	52.65	7.09
NH_3_ (mg/L)	0.29	4.42	1.91	1.48
NO_3_^−^ (mg/L)	73.98	2105.56	510.83	543.20

TEMP: sea surface temperature, COND: conductivity, SAL: salinity, TDS: total dissolved solids, LDO: luminescent dissolved oxygen, TRANS: transparency, TURB: turbidity, PHYCO: phycocyanins, CHLORO: chlorophyll, ORP: oxidation reduction potential, NH_4_^+^: Ammonium, NH_3_: Ammonia, and NO_3_^−^: Nitrate.

**Table 2:** Comparison of the concentrations of potentially toxic and essential elements (mg/L) in the water from the coast of Callao Bay with national and international regulations.

Elements	Peruvian Standard		USEEPA		WHO	Callao Bay			
	II-C3	IV-E3	MCC	CCC		P1	P2	P3	P4
Potentially toxic elements									
Ag	0.007		0.002		0.005	0.003 * ± 0.002	0.004 * ± 0.002	0.003 * ± 0.001	0.004 * ± 0.001
As	0.05	0.036	0.069	0.036	0.01	0.009 ± 0	0.009 ± 0	0.009 ± 0	0.009 ± 0.011
Ba					1.3	4.737 * ± 0.002	5.080 * ± 0.001	4.810 * ± 0.008	5.700 * ± 0.008
Cd		0.009	0.040	0.009	0.003	0.002 ± 0.001	0.002 ± 0.001	0.002 ± 0	0.002 ± 0.001
Cr	0.05	0.05			0.05	0.003 ± 0.002	0.006 ± 0.002	0.005 ± 0.003	0.005 ± 0.002
Hg	0.002	0.0001	0.002	0.001	0.006	0.001 * ± 0	0.001 * ± 0	0.001 * ± 0	0.001 * ± 0
Ni	0.052	0.008	0.074	0.008	0.07	0.015 * ± 0.008	0.023 * ± 0.009	0.014 * ± 0.010	0.030 * ± 0.010
Pb	0.03	0.008			0.01	0.032 * ± 0.007	0.055 * ± 0.031	0.068 * ± 0.025	0.043 * ± 0.011
Essential elements									
Bi						0.014 ± 0.012	0.020 ± 0.014	0.008 ± 0.005	0.008 ± 0.002
Co						0.003 ± 0.001	0.004 ± 0.002	0.006 ± 0.007	0.003 ± 0.003
Cu	0.05	0.05	0.005	0.008	2	0.007 ± 0.002	0.005 ± 0.005	0.007 ± 0.002	0.011 ± 0.004
Fe					2	0.158 ± 0.072	0.107 ± 0.051	0.120 ± 0.051	0.017 ± 0.029
P		0.06				0.165 * ± 0.078	0.156 * ± 0.035	0.168 * ± 0.067	0.006 ± 0.023
Mn					0.4	0.006 ± 0.004	0.003 ± 0.002	0.004 ± 0.003	0.004 ± 0.004
Se			0.290	0.071	0.01	0.0250 * ± 0.020	0.025 * ± 0.024	0.020 * ± 0.014	0.025 * ± 0.035
Si *	0.700					1.2259 * ± 0.250	1.462 * ± 0.490	1.364 * ± 0.261	1.010 * ± 0.193
Sn					0.01	0.015 * ± 0.003	0.016 * ± 0.007	0.014 * ± 0.009	0.014 * ± 0.003
Zn	0.12	0.081	0.090	0.081	1	0.0128 ± 0.003	0.035 ± 0.034	0.016 ± 0.006	0.010 ± 0.095

* Element concentrations above the quality standards of some regulations in comparison. P1: the Naval School; P2: IMARPE; P3: Callao Pier; P4: San Lourenço Island. Peruvian Standard: II-C3 = Category 2: Extraction, crops, and other coastal and continental marine activities. C3: Marine port, industrial, or sanitation activities in coastal marine waters. IV-E3 = Category 4: Conservation of the aquatic environment. E3: Marine ecosystems. USEPA: Environmental Protection Agency. MCC: maximum concentration criterion, which is an estimate of the highest concentration of a material in surface water that an aquatic community can be briefly exposed to without resulting in an unacceptable effect. CCC: continuous concentration criterion, which is an estimate of the highest concentration of a material in surface water to which an aquatic community can be exposed indefinitely without producing an unacceptable effect. World Health Organization (WHO) Guidelines for Drinking Water Quality: Recommendations [66].

**Table 3:** Comparison of the concentration of potentially toxic and essential elements (mg/kg) in the sediment from the Bay of Callao with international regulation.

Elements	Minimum	Maximum	Mean	Standard Deviation	USEPA (Not Polluted)
Potentially toxic elements					
Ag	0.10	2.00	1.09	0.70	
Al	7909.00	19,124.00	13,259.10	3481.15	
As	14.45	170.80	45.55	48.40	<3
Ba	52.87	149.40	103.42	33.43	
Be	0.25	0.49	0.36	0.08	
Cd	1.45	9.53	5.24 *	3.31	3.00
Cr	11.61	29.02	19.98	7.59	<25
Hg	0.15	1.80	0.64	0.51	
Li	13.20	28.60	20.94	5.55	
Ni	4.88	14.38	9.02	3.50	<20
Pb	26.40	275.10	122.95 *	99.41	<40
Sb	0.60	3.10	1.36	0.78	
Sr	51.90	359.50	212.44	94.40	
Ti	480.80	988.20	666.92	150.19	
Essential elements					
B	16.39	41.94	28.31	9.58	
Cu	19.11	298.20	98.96	88.14	<25
Co	4.47	10.31	6.89	2.07	
Ca	11,675.00	55,989.00	34,438.90	13,327.13	
Ce	12.68	21.10	16.61	2.69	
Fe	12,868.00	28,566.00	20,727.60	4889.61	
P	1177.00	1632.00	1396.00	123.36	
K	1376.00	3802.00	2559.70	871.75	
Mg	4866.00	11,480.00	8536.70	2232.16	
Mn	166.20	421.30	280.40	74.93	<300
Mo	0.66	7.29	3.26	2.65	
Na	5098.00	26,150.00	14,156.40	7566.34	
Va	35.45	70.17	54.57	11.29	
Zn	87.96	1131.00	391.43	347.95	

* Element concentrations above the quality standards of some regulations in comparison. USEPA: United States Environmental Protection Agency guidelines for sediments.

**Table 4:** Comparison of the concentrations of potentially toxic and essential elements (mg/kg) in the muscle tissue of *Sciaena deliciosa* from the Bay of Callao, Peru.

Elements	WHO/FAO (CODEX)	FAO (n° 66/2003)	Callao Bay			
			P1	P2	P3	P4
Potentially toxic elements						
Ag			0.13 ± 0.04	<0.1 ± 0.01	<0.1 ± 0.01	<0.1 ± 0.01
As	0.5	0.5	5.44 * ± 4.22	3.52 * ± 1.09	2.68 * ± 2.39	3.27 * ± 2.57
Al			14.09 ± 6.97	26.05 ± 15.66	12.53 ± 4.45	12.71 ± 9.02
Ba			0.55 ± 0.54	2.76 ± 2.32	0.75 ± 0.41	0.38 ± 0.22
Cd	0.5	0.05	<0.15	<0.15	<0.15	<0.15
Cr		1.0	0.39 ± 0.24	1.36 * ± 1.91	0.64 ± 0.17	0.20 ± 0.77
Hg	0.5	0.2	0.22 * ± 0.18	0.12	0.25 * ± 0.25	0.14 ± 0.03
Li			0.80 ± 0.10	0.94 ± 0.21	0.95	1.13 ± 0.1
Pb	0.3	0.2	5.81 * ± 8.39	1.56 *	0.98 *	0.72 * ± 0.79
Ni		1.0	0.88 ± 0.45	0.46	0.51	0.46 ± 0.15
Sb			<0.6	0.75 ± 0.1	0.71 ± 0.11	1.43
Sr			10.28 ± 4.35	13.53 ± 6.54	17.77 ± 9.67	21.88 ± 6.85
Ti			0.19 ± 0.10	0.97 ± 0.39	0.57	0.19 ± 0.16
Essential elements						
B			2.28 ± 0.55	1.99 ± 0.60	1.99 ± 0.49	1.50 ± 0.55
Cu			2.39 ± 1.55	1.52 ± 0.38	1.53 ± 0.49	1.94 ± 0.81
Ca			2132 ± 1119.66	3148.33 ± 1447.73	3943.5 ± 1884.44	4239 ± 1483.94
Fe			63.62 ± 16.52	117.42 ± 111.39	47.34 ± 1.77	60.96 ± 43.23
P			10,212.75 ± 954.19	10,761.33 ± 898.76	11,785.5 ± 1236.73	11,442 ± 1029.89
K			16,430.25 ± 1024.38	16,246 ± 761.27	17,164.5 ± 420.73	14,993 ± 735.46
Mg			1378.25 ± 32.48	1377.33 ± 88.64	1429.5 ± 71.42	1310 ± 64.18
Mn			0.93 ± 0.14	1.66 ± 0.90	1.05 ± 0.27	1.18 ± 0.43
Na			3426.75 ± 1114.48	3654 ± 1015.99	2907 ± 1760.69	2622 ± 1297.05
Se			3.14 ± 1.14	1.49 ± 0.04	2.85 ± 1.9	1 ± 1.03
Zn			19.19± 3.74	16.44 ± 1.36	17.54 ± 2.36	18.86± 2.49

* Element concentrations above the quality standards of some regulations in comparison. P1: the Naval School; P2: IMARPE; P3: Callao Pier; P4: San Lourenço Island. WHO/FAO Codex Alimentarius Commission. Food and Agriculture Organization (FAO) Heavy Metal Regulations—Faolex. Legal Notice No. 66/2003. 2003. The Tukey test was performed (*p* < 0.05) for mean comparison; however, there was no significant difference between the groups.

**Table 5:** Comparison of the concentrations of potentially toxic and essential elements (mg/kg) in the liver tissue of *Sciaena deliciosa* from the Bay of Callao, Peru.

Elements	WHO/FAO (CODEX)	FAO (n° 66/2003)	Callao Bay			
			P1	P2	P3	P4
Potentially toxic elements						
Ag			0.50 ± 0.1	<0.2	0.29 ± 0.03	<0.2
As	0.5	0.5	1.47 *	<0.92 *	4.22 * ± 4	<0.92 *
Al			9.31 ± 2.85	17.67 ± 11.00	3.31 ± 0.84	3.61 ± 0.85
Ba			0.24 ± 0.12	0.59 ± 0.1	0.14 ± 0.1	<0.13 ± 0.1
Cd	0.5	0.05	0.92 * ± 0.09 ab	0.47 * ± 0.30 b	0.71 * ± 0.39 b	1.67 * ± 0.26 a
Cr		1.0	0.20 ± 0.01	<0.16	0.20 ± 0.1	0.29 ± 0.1
Hg	0.5	0.2	0.19 ± 0.13	0.26 * ± 0.16	0.13 ± 0.1	0.1 ± 0.1
Li			0.30 ± 0.10	0.40 ± 0.10	0.20 ± 0.20	0.20 ± 0.13
Pb	0.3	0.2	2.10 * ± 1.20	<0.3	1.37 * ± 0.10	<0.3
Ni		1.0	1.40 *	<0.64	<0.64	<0.64
Sb			<0.6	<0.6	<0.6	<0.6
Sr			2.53 ± 0.86	2.97 ± 0.29	2.77 ± 2.21	6.11 ± 1.12
Ti			0.20 ± 0.05	0.76 ± 0.5	0.1 ± 0.1	<0.12
Essential elements						
B			1.93 ± 0.23	1.48 ± 0.17	1.50 ± 0.51	1.67 ± 0.30
Cu		0.6	13.43 * ± 5.46	5.66 * ± 2.92	12.92 * ± 9.38	14.61 * ± 5.92
Ca			303.15 ± 80.26	483.55 ± 53.81	358.18 ± 262.22	896.10 ± 132.10
Fe			236.25 ± 2.33	215.90 ± 42.57	290.11 ± 37.32	250.80 ± 27.41
P			4585 ± 524.67	4068.5 ± 522.55	4741.5 ± 252.44	6259 ± 433.22
K			4586.5 ± 508.41	4218.50 ± 828.02	4889.5 ± 293.45	5954 ± 543.29
Mg			451.70 ± 30.41	448.25 ± 49.99	514.87 ± 227.6	547.40 ± 102.66
Mn			2.37 ± 0.47	2.19 ± 1.05	1.94 ± 0.77	2.31 ± 0.76
Na			2953.5 ± 218.5	2642 ± 830.14	2837.5 ± 1590.28	2961 ± 879.64
Se			3.50 ± 2.12	3 ± 1	3.42 ± 1.1	1.4 ± 1.12
Zn			45.53 ± 2.76	36.92 ± 5.05	49.15 ± 7.45	53.63 ± 5.09

* Element concentrations above the quality standards of some regulations in comparison. P1: the Naval School; P2: IMARPE; P3: Callao Pier; P4: San Lourenço Island. WHO/FAO Codex Alimentarius Commission. Food and Agriculture Organization (FAO) Heavy Metal Regulations—Faolex. Legal Notice No. 66/2003. 2003. The Tukey test was performed (*p* < 0.05) for mean comparison; however, there was a significant difference only for Cd, where “a” and “b” alone denote significant differences in relation to other groups.

**Table 6:** Bioconcentration factor (BCF) index in *Sciaena deliciosa* from the Bay of Callao, Peru.

Elements	Muscle (Times)	Liver (Times)
Phosphorus	87,056	42,493
Iron	1.35	3219
Zinc	1.33	3149
Manganese	566	-
Arsenic	442	-
Mercury	362	1509

**Table 7:** Assessment of the health indexes for *Sciaena deliciosa* from the Bay of Callao, Peru, for adults.

Adults	EDI P1	EDI P2	EDI P3	EDI P4	Oral Reference Dose (RfD) (mg/kg/day)	THQ P1	THQ P2	THQ P3	THQ P4	CR P1	CR P2	CR P3	CR P4
Potentially toxic metals													
Ag	0.0020	0.0028	0.0021	0.0028	0.005	0.2968	0.4240	0.3180	0.4240				
As	0.0065	0.0064	0.0064	0.0064	0.0003	16.2533	15.9000	15.9000	15.9000	0.000979	0.000958	0.000958	0.000958
Cd	0.0014	0.0014	0.0014	0.0014	0.001	1.0600	1.0600	1.0600	1.0600				
Cr	0.0025	0.0043	0.0036	0.0036	0.0015	1.2367	2.1200	1.7667	1.7667				
Hg	0.0002	0.0007	0.0007	0.0007	0.0005	0.3180	1.0600	1.0600	1.0600				
Pb	0.0226	0.0391	0.0483	0.0305	0.004	4.2135	7.2875	9.0100	5.6975	0.000191	0.000331	0.000410	0.000259
Ni	0.0109	0.0163	0.0099	**0.0213**	0.020	0.4055	0.6095	0.3710	0.7950				
Essential elements													
Cu	0.0053	0.0036	0.0050	0.0078	0.40	0.0098	0.0066	0.0093	0.0146				
Fe	0.1120	0.0760	0.0852	0.0121	0.80	0.1045	0.0709	0.0795	0.0113				
Mn	0.0046	0.0021	0.0028	0.0028	0.14	0.0246	0.0114	0.0151	0.0151				
Se	0.0178	0.0178	0.0142	0.0178	0.005	**2.6500**	**2.6500**	**2.1200**	**2.6500**				
Zn	0.0091	0.0249	0.0114	0.0071	0.3	0.0226	0.0618	0.0283	0.0177				
HI						**26.5953**	**31.2617**	**31.7379**	**29.4118**				

P1: the Naval School; P2: IMARPE; P3: Callao Pier; P4: San Lourenço Island. EDI: estimated daily intake (mg/kg), THQ: target hazard quotient, HI: hazardous index, CR: carcinogenic risk. From: US Environmental Protection Agency/USEPA [69].

**Table 8:** Assessment of health indexes in *Sciaena deliciosa* from the Bay of Callao, Peru, for children.

Children	EDI P1	EDI P2	EDI P3	EDI P4	Oral Reference Dose (RfD) (mg/kg/day)	THQ P1	THQ P2	THQ P3	THQ P4	CR P1	CR P2	CR P3	CR P4
Potentially toxic elements													
Ag	**0.009324**	**0.01332**	**0.00999**	**0.01332**	0.005	0.1120	0.1600	0.1200	0.1600				
As	**0.030636**	**0.02997**	**0.02997**	**0.02997**	0.0003	**6.1333**	**6.0000**	**6.0000**	**6.0000**	**0.004595**	**0.004495**	**0.004495**	**0.004495**
Cd	**0.00666**	**0.00666**	**0.00666**	**0.00666**	0.001	0.4000	0.4000	0.4000	0.4000				
Cr	**0.011655**	**0.01998**	**0.01665**	**0.01665**	0.0015	0.4667	0.8000	0.6667	0.6667				
Hg	**0.000999**	**0.00333**	**0.00333**	**0.00333**	0.0005	0.1200	0.4000	0.4000	0.4000				
Pb	**0.105894**	**0.18315**	**0.22644**	**0.14319**	0.004	**1.5900**	**2.7500**	**3.4000**	**2.1500**	0.000900	0.001557	0.001925	0.001217
Ni	**0.050949**	**0.07659**	**0.04662**	**0.0999**	0.020	0.1530	0.2300	0.1400	0.3000				
Essential elements													
Cu	0.024642	0.01665	0.02331	0.03663	0.40	0.0037	0.0025	0.0035	0.0055				
Fe	0.525474	0.35631	0.3996	0.05661	0.80	0.0395	0.0268	0.0300	0.0043				
Se	0.08325	0.08325	0.0666	0.08325	0.005	**1.0000**	**1.0000**	0.8000	**1.0000**				
Mn	0.021645	0.00999	0.01332	0.01332	0.14	0.0093	0.0043	0.0057	0.0057				
Zn	0.042624	0.11655	0.05328	0.0333	0.3	0.0085	0.0233	0.0107	0.0067				
HI						**10.0360**	**11.7969**	**11.9765**	**11.0988**				

P1: the Naval School; P2: IMARPE; P3: Callao Pier; P4: San Lourenço Island. EDI: estimated daily intake (mg/kg), THQ: target hazard quotient, HI: Hazardous Index, CR: Carcinogenic Risk. From: US Environmental Protection Agency/USEPA [66].

## Data Availability

Data are contained within the article.
